# Energetics of hydrogen adsorption and diffusion for the main surface planes and all magnetic structures of γ-iron using density functional theory

**DOI:** 10.1039/d1ra04999b

**Published:** 2021-08-27

**Authors:** Urslaan K. Youhan, Sven P. K. Koehler

**Affiliations:** School of Materials, The University of Manchester Manchester M13 9PL UK; Department of Natural Sciences, Manchester Metropolitan University Manchester M1 5GD UK s.koehler@mmu.ac.uk

## Abstract

In this study, we calculated the energetics of hydrogen atoms adsorbing on and diffusing into the first few layers of γ-Fe for the (100), (110) and (111) surfaces and for the non-magnetic (NM), ferromagnetic (FM), and antiferromagnetic single (AFM1) and double layer (AFMD) structures. These studies are relevant as they atomistically simulate the early stages of hydrogen embrittlement in steels. We employed density functional theory to establish adsorption sites and energies for each plane and the minimum energy pathways for diffusion through the first few layers with associated activation barriers. Adsorption energies for all cases vary between ∼3.7 and 4.4 eV, and the energy barriers to diffusion in the bulk region vary between ∼0.2 and 1.2 eV for the twelve cases, with the highest and lowest bulk diffusion barriers occurring in the NM(111) and the FM(100) case, respectively. We conclude that the texturing of steels in order to expose certain cleavage planes or magnetic structures can decrease the likelihood of hydrogen embrittlement.

## Abstract 2

Calculations of adsorption and diffusion energies of hydrogen on and through iron relevant to hydrogen embrittlement.

## Graphical abstract

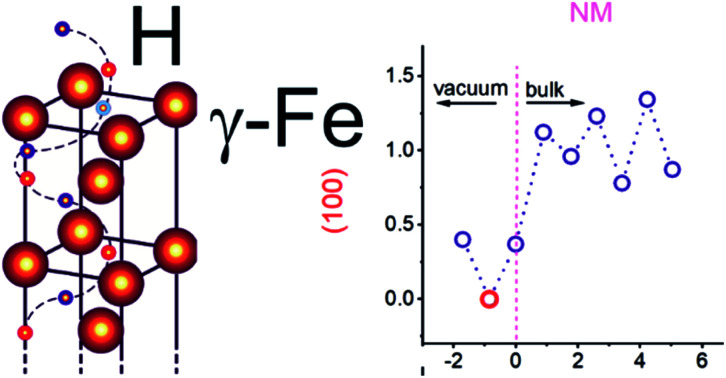

## 1. Introduction

This publication concludes a series of papers in which we modelled the adsorption of oxygen and hydrogen on bcc iron,^1,2^ and, more relevant to the current paper, the hydrogen adsorption on and sub-surface diffusion through γ-Fe.^3,4^ The last two publications focussed on austenitic (stainless) steel as opposed to ferritic or ferritic-martensitic steels, in particular the face-centred cubic (fcc) austenitic steel phase, termed austenite or γ-Fe. In ref. 3, we focussed on the different crystal planes (100), (110), and (111) but all for the non-magnetic case. In ref. 4, we limited ourselves to the (100) surface but treated the four different magnetic structures, namely non-magnetic (NM), ferromagnetic (FM), and antiferromagnetic single (AFM1) and double layer (AFMD) structures. This work completes this endeavour by presenting the energetics for all twelve combinations of crystal cuts and magnetic structures, *i.e.* all twelve combinations of (100), (110), and (111) surfaces for all magnetic structures NM, FM, AFM1 and AFMD.

As there is already a large body of work concerned with hydrogen diffusion on and through iron for the body-centred cubic α-phase,^2,5–8^ we investigate here the face-centred cubic (fcc) phase of iron, γ-Fe. This phase is stable in the range of temperatures of 1185–1665 K,^9^ but can also be present as a metastable grain at lower temperatures by adding γ-stabilising alloying elements such as nickel, manganese, carbon or nitrogen.^10,11^ Such alloys are also used to form austenitic steels which find application, amongst others, in the nuclear industry,^12,13^ the offshore industry,^14^ and the automotive industry.^15–18^ The complex magnetic behaviour of the γ-phase can also be tuned by varying the amount of alloying elements, thus changing the magnetic ground state and the critical temperature of γ-Fe,^19,20^ making this field relevant to *e.g.* magnetoelectronics,^21^ biomedicine,^22^ and also in steel components close to the D–T plasma of magnetically-confined fusion reactors.^23^

The three magnetic phases of fcc γ-Fe are considered in the collinear approximation: the ferromagnetic (FM-↑↑↑↑…) phase, as well as multi-layered anti-ferromagnetic phases, namely the single (AFM1-↑↓↑↓…) and double (AFMD-↑↑↓↓…) layer phases.^24–26^ Tsunoda report that the double-layer structure is the energetically most favourable.^27^ This magnetic behaviour is not limited to the bulk, but can also be observed experimentally on surfaces,^28^ as well as modelled.^29^ The magnetic ordering in γ-Fe is due to the interaction of itinerant d-electrons which may move between atoms when interacting with interstitial atoms. Since one of the aims in this project is to trace the minimum energy path (MEP) of H atoms diffusion through γ-Fe, the magnetic state of the metal may have an effect on the interstitial diffusion pathway and energetics. Open grain boundaries in non-magnetic fcc Fe, such as Σ11, offer additional H trapping sites and also provide diffusion pathways for hydrogen with an energy barrier of 0.7 eV based on DFT calculations.^30^

Our overall goal to gain an atomistic understanding of hydrogen diffusion in γ-Fe is driven by the issue of hydrogen embrittlement (HE), a process discovered by Johnson in 1875,^31^ critical to the degradation of iron materials and structures. HE affects the integrity of materials such as advanced steels,^32,33^ and nickel-based alloys.^34,35^ This can lead to material failure with potentially catastrophic consequences, and even the cost of preventing such failures – once HE has been detected – can be significant, such as the failure of steel bolts due to HE in a single high-rise office block, costing over £6 M.^36^

In the HE process, hydrogen atoms diffuse into and accumulate in the bulk structure of metals, resulting in its brittleness. The two principal mechanisms proposed for HE are: HELP (Hydrogen Enhanced Localised Plasticity), in which hydrogen lowers the activation energy for dislocation such that highly-deformed localised regions are created,^37,38^ and HEDE (Hydrogen Enhanced DEcohesion), in which the hydrogen reduces the interatomic cohesive forces and favours the cleavage of planes along grain boundaries.^39,40^ On smaller length-scales or a more microscopic level, hydrogen initially physisorbs onto the metal surface,^41,42^ where the molecules dissociate and bond on the adsorption site with the lowest energy. The atoms may recombine, or due to the higher bulk diffusion constants may enter the bulk as hydrogen atoms,^43^ where they move along the MEP before accumulating on defect sites.

**Fig. 1:**
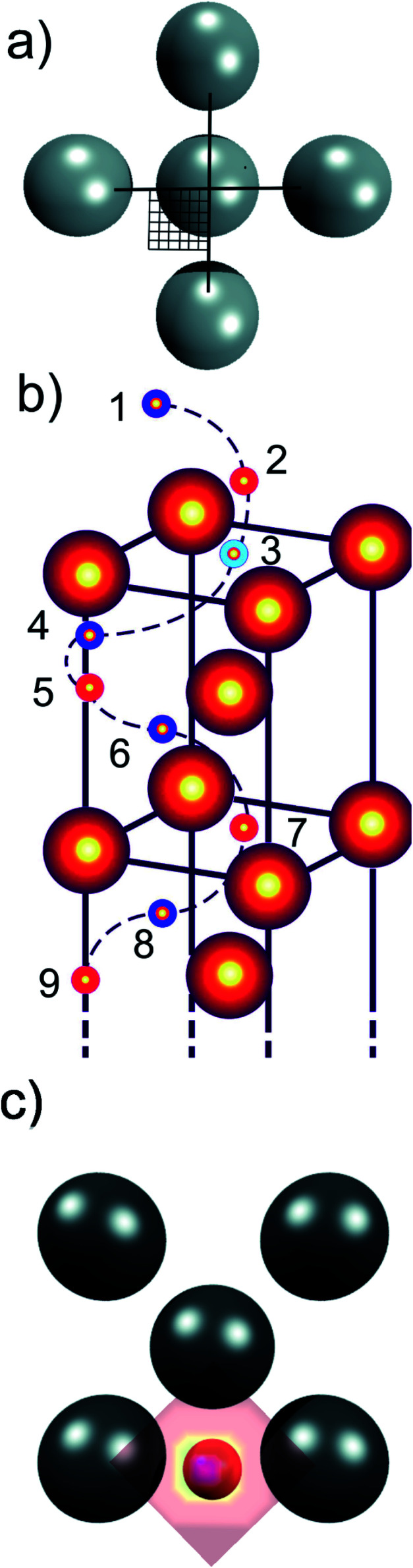
(a) Illustration of the 36 points at which hydrogen atoms were placed and the geometry optimised and energy calculated, delivering information about the PES for the whole surface unit cell due to symmetry considerations, in this case for the (100) plane; (b) illustration of the nine depth at which a 2D grid as shown in (a) was spanned to identify the minimum energy path for a reduced (100) cut. (c) 2D PES showing the preferred adsorption geometry of hydrogen (in red) just above the (100) surface for the ADMD case, where red colour is the energy maximum, and purple (hidden under the red H atom) the minimum.

One of the advantages of computational methods such as condensed-phase density functional theory (DFT) is that it is possible to simulate chemical processes at an atomistic level. DFT has previously been used to model hydrogen diffusion into subsurfaces,^44,45^ and through bulk metal.^46^ We have hence chosen to apply DFT to model hydrogen diffusion in γ-Fe, to investigate the potential energy surface (PES) that governs diffusion, and to elucidate the early stages of HE to gain a detailed understanding of the on-surface adsorption and sub-surface diffusion process through γ-Fe. We have here optimised the energies of a single H atom at a relatively large number of points on a regularly-spaced 2D grid covering the surface unit cell (see Fig. 1a) at various heights above the Fe surface and below in the bulk (see Fig. 1b), *i.e.* for every selected height *z*, we calculated a 2D PES as a function of the *x* and *y* coordinates parallel to the surface. We can thus determine the minimum energy sites along which the H atoms moves, extract the MEP for H diffusion into the bulk, and create energy profiles that allow us to obtain the activation energy and bulk diffusion barrier along the way. This is fundamental for a better understanding of the earliest stages of hydrogen embrittlement, and to device routes of how to minimise the likelihood of hydrogen embrittlement.

## 2. Computational methods

The potential energy surfaces for hydrogen adsorption on the surface and diffusion into sub-surfaces of γ-Fe were calculated for the (100), (110) and (111) surfaces, using density functional theory (DFT). The non-magnetic (NM), ferromagnetic (FM), anti-ferromagnetic single (AFM1) and double (AFMD) layer magnetic states were considered for each surface. The NM state was modelled using non-spin polarised DFT, while the FM, AFM1 and AFMD states were incorporated in the model using spin-polarisation in the collinear approximation. The Vienna *ab initio* simulation package (VASP) was employed,^47^ and a plane-wave basis set with 3D periodic boundary conditions described electronic interactions. The exchange and correlation effects were included using the generalised-gradient approximation (GGA), *via* the Perdew–Burke–Ernzerhof (PBE) functional,^48^ and the projector augmented-wave (PAW) approximation describes the interaction between the ionic core and valence electrons.^49^

A seven Fe layer slab model was used to model surfaces and bulk of γ-Fe, with the three bottom layers frozen to represent the bulk region below the surface, while the Fe atoms in the top four layers were allowed to relax in the *xy* plane. A vacuum spacing of 20 Å and a cutoff energy of 400 eV were found to sufficiently converge the total energy of the system. The Monkhorst–Pack algorithm was used with a grid size of 7 × 7 × 1 for the (100), (110) and (111) surfaces.^50^ The Methfessel Paxton method of order *N* = 1 with width 0.1 eV was used to apply electronic smearing.^51^ The slabs were minimised using the conjugate gradient method,^52^ until forces were within 10^−5^ eV Å^−1^. The energies of all atoms were converged to within 10^−6^ eV.

A large number of hydrogen positions were sampled within the slab using a mesh grid to accurately describe the potential energy surface. A quarter of the 2 × 2 surface unit cell was sampled in the *x*–*y* plane parallel to the surface with the *z* direction constrained, using a tight 6 × 6 uniform grid on the domain *x*,*y* ∈ [0,0.5], in fractional coordinates (see Fig. 1a). Due to symmetry considerations, we only needed to sample a quarter of the surface unit cell but obtained, due to symmetry considerations, 144 points for every depth (along *z*), or 1296 optimised energies for each slab. These nine heights along the *z* direction consist of four meshes (each with 144 sampled points) at the height of the first four Fe layers (layers 3, 5, 7, 9), three further meshes located centrally in-between these Fe layers (layers 4, 6, 8), and finally two meshes above the surface to simulate H adsorption (layers 1 and 2) (see Fig. 1b). The energies at each point, *E*, were calculated *via* the relation

1 *E* = *E*_slab+H_ − (*E*_slab_ + *E*_H_)

where *E*_slab+H_ is the energy of the H-containing slab, and *E*_H_ is the ground state energy of a single free H atom in a 10 × 10 × 10 A^3^ box. The energies were then calculated relative to the global minimum of the entire slab, which was set to zero energy. We favoured this grid method over the nudged elastic band (NEB) method;^53^ while the latter may give a better representation of the MEP by probing a number of images between the minimum and maximum, our grid method has the advantage of probing the overall energy landscape not only close to the MEP, but in the entire crystal. It was also shown for hydrogen diffusion on various metal surfaces that differences in the activation barrier obtained from studies based on the PES and the NEB method did not vary by more than 0.01 eV.^54^

## 3. Results and discussions

**Fig. 2:**
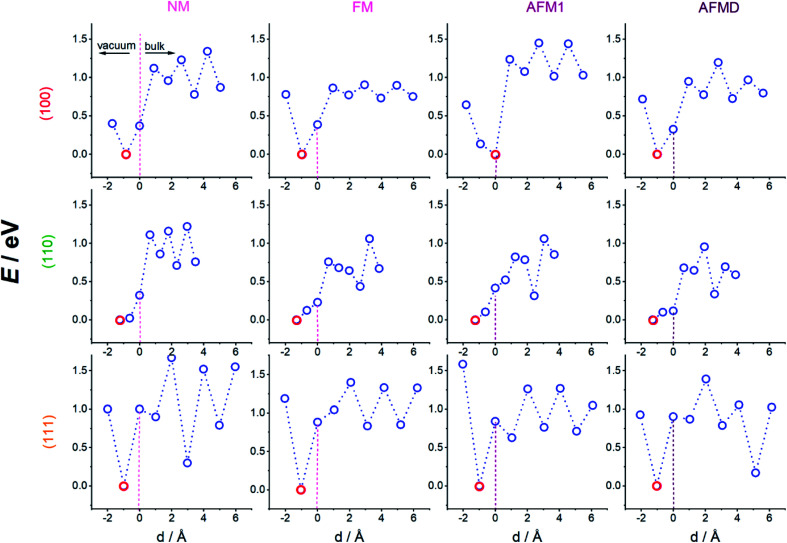
Potential energy surfaces along the minimum energy path for hydrogen adsorbing onto and diffusing into single-crystal iron for the three surface planes as indicated on the left, and the four magnetic structures indicated above each column. Hydrogen diffuses from left (above surface) to right (into the bulk), lowest energy point each highlighted in red.

Since the effects of hydrogen diffusion on the exact geometry of the unit cells and the magnetic properties were already discussed in previous publications,^3,4^ we focus here mainly on the energetics of the hydrogen adsorption and diffusion process for the twelve cases which are a combination of three different surface planes, namely (100), (110), and (111), and four different magnetic structures, namely NM, FM, AFM1, and AFMD, for γ-Fe. The numerical results are summarised in Table 1 and the respective potential energy surfaces depicted in Fig. 2, in which we show the energy at two heights above the surface, at the height of the first layer of Fe atoms (dotted vertical line at *d* = 0 Å), and at six heights below the surface (three each in a plane of Fe atoms, and between planes). It can be seen that the magnetic structure does not have an influence on the adsorption site, but naturally the surface plane does. Hydrogen adsorbs on the 4f site for the (100) and the (110) surface, and on the 3f site for the (111) cut. The distance between the adsorbed hydrogen atoms and the outermost plane of iron atoms does also not seem to depend on the magnetic structure, but on the adsorption site and hence surface plane, with the hydrogen atoms adsorbing on the 4f site for the (110) surface only being 0.6 to 0.7 Å above the surface plane, while the hydrogen atoms in the 4f site for the (100) surface adsorb 0.9–1.0 Å above the surface plane, and in the 3f site for the (111) plane 1.0 ± 0.1 Å above the outermost Fe atoms.

**Table 1:** Hydrogen adsorption height (*d*_H-surf_, in Å), adsorption energies (*E*_ads_), total diffusion barriers (*E*_total_barrier_), and bulk diffusion energies (*E*_bulk_, all in eV) for the twelve different surface planes and magnetic cases for γ-Fe. The magnetic moments of the four different magnetic structures are symbolised in the first row for the non-magnetic (NM), ferromagnetic (FM), antiferromagnetic single (AFM1) and double layer (AFMD), surface planes indicated in pink in the first column

fcc-Fe (adsorption site)		NM	FM	AFM1	AFMD
100 (4f)	*d*_H-surf_/Å	0.9	1.0	0.9	1.0
	*E*_ads_/eV	4.07	4.12	4.03	4.05
	*E*_total_barrier_/eV	1.3	0.9	1.4	1.2
	*E*_bulk_/eV	0.6	0.2	0.4	0.5
110 (4f)	*d*_H-surf_/Å	0.6	0.7	0.6	0.7
	*E*_ads_/eV	3.92	4.36	4.28	3.76
	*E*_total_barrier_/eV	1.2	1.1	1.1	1.0
	*E*_bulk_/eV	0.5	0.6	0.7	0.6
111 (3f)	*d*_H-surf_/Å	1.0	1.0	1.0	1.1
	*E*_ads_/eV	4.05	4.27	4.18	4.35
	*E*_total_barrier_/eV	1.7	1.4	1.3	1.4
	*E*_bulk_/eV	1.2	0.5	0.6	0.9

The adsorption energies do not vary significantly but are around 4 eV in all cases. This means that hydrogen atoms are almost equally likely to adsorb on a sample of γ-Fe no matter which surface is exposed. The likelihood of the initial step of hydrogen embrittlement, the adsorption of H onto the surface, occurring can hence not be altered by structuring the grains in a polycrystalline iron sample.

By connecting the minima of the hydrogen positions at the nine different heights, we established minimum energy paths (MEP) for the hydrogen adsorbing onto and diffusing into the Fe. An example of such a MEP is shown in Fig. 1 b, and the corresponding PESs for all 12 cases are shown in Fig. 2, displaying the potential energy as a function of depth. The MEPs are independent of magnetic cases for the same surface plane,^4^ but naturally differ for the three different surface planes.^3^ The overall barrier for the hydrogen to penetrate into the bulk (*E*_total_barrier_, calculated as the difference between the global maximum and minimum on our PESs) is broadly between 1.0 and 1.4 eV, but texturing polycrystalline austenitic steel samples such that (111) surfaces are exposed predominantly might prevent hydrogen embrittlement to some extent.^55^

It can be seen from Fig. 2 that the local minima for the (100) surface are located in the plane of Fe atoms for all sub-surfaces (at the octahedral sites, not shown), those planes are located at the odd-numbered heights in Fig. 1, *i.e.* 3, 5, 7, and 9, while position 1 is outside the crystal. On the other hand, for the (111) surface, the local minima are between the close-packed Fe planes (at depth positions 4, 6, and 8), with the local maxima in the planes containing Fe atoms. The non-magnetic (110) case is similar to all (100) cases, *i.e.* minima in Fe planes alternatively at long and short-bridge sites, but interestingly, the PES for the FM, AFM1 and AFMD cases are much more complex and do not display such a regular pattern. This is likely because the s orbital electron in hydrogen interact with the itinerant d electrons of Fe, and *vice versa*, and hence the local Fe magnetism affects the energetics of hydrogen diffusion through the fcc Fe lattice.

Once the H atoms move past the outermost Fe layer, the H atoms move from one minimum energy position to the next. The (sub-surface or) bulk diffusion barriers (*E*_bulk_, calculated as the difference between the maximum and minimum energy for all position below the surface, *i.e.* at heights 4–9) vary broadly between 0.5 and 0.6 eV, which is too high an energy to be purely thermally activated at moderate temperatures. The notable exception is the much lower bulk diffusion barrier through ferromagnetic (100) γ-Fe of 0.2 eV, which means that at ∼2300 K hydrogen embrittlement can be considered diffusion controlled. In this case a combination of low bulk diffusion and low total barrier are making hydrogen embrittlement much more likely. On the opposite end of the scale, both the bulk diffusion barrier as well as the total barrier are highest for the non-magnetic and double-layer close-packed Fe(111) structures, making these structures less susceptible to hydrogen embrittlement. The fact that both non-magnetic as well as double-layer structures are affected points to a magnetic ordering effect, *i.e.* the greater order in the FM and AFM1 structures facilitate lower barrier H diffusion.

Bulk diffusion energies in austenitic steels (containing traces of alloying elements) have been measured previously, and our values (0.5–0.6 eV) compare favourably with experimentally determined diffusion barriers (∼0.5–0.7 eV).^56–68^

It can also be seen from Fig. 2 that almost all magnetic states of the (100) and (111) surface have an energy minimum at the adsorption site, but such a stable adsorption state does not exist for the (110) cut. Nevertheless, adsorption on and penetration into the Fe crystal are still endothermic processes for all magnetic states of the (110) cut.

Comparing overall activation barriers (*E*_total_barrier_) with the bulk diffusion barriers (*E*_bulk_), it is clear that it takes around double the energy to initially embed the hydrogen into each surface, whilst bulk diffusion requires relatively less energy; the initial penetration of the H atom into the Fe slab hence seems to be the rate-determining step.

The regular pattern of peaks and troughs in the bulk PES, at least for the (100) and (111) case, are noticeably broken for the AFMD magnetic structure, which is likely due to the switch in the spin direction between the first and second (same), and third and fourth layers. However, despite this change in energy profile, the movement of H from one octahedral site to the next in case of the (100) surface persists for all four magnetic cases, showing that the magnetism does not have a noticeable effect on the exact MEP, *i.e.* the minimum and maximum energy sites along which the H travels, but instead magnetism affects the actual energies to a greater extent.

It is worth stressing that we have not considered quantum tunnelling effects in our calculations, but given the low mass of hydrogen, these could feasibly change the results. Tunnelling would lead to higher diffusion coefficients despite the classical barrier remaining constant, as the H would tunnel through the barrier. However, whereas the magnitude of tunnelling would stay constant over the whole temperature range, its contribution to the overall diffusion coefficient would decrease at increasing temperatures as classical trajectories over the barrier dominate,^69–71^ hence comparison of the barriers calculated here with the diffusion coefficients measured at room temperature is legitimate.

These DFT results considering the four magnetic states and three crystal cuts have implications for HE of Fe-based alloys. If an austenitic alloy is selected which has ferromagnetic ordering of the Fe atoms, then the H atoms more readily diffuse through the bulk than for the other magnetic states, especially for FM(100). As a result, ferromagnetic alloys are most susceptible to hydrogen embrittlement.

## 4. Conclusions

We employed spin-polarised density functional theory to investigate hydrogen diffusion through the three major planes in γ-Fe, namely the (100), (110) and (111) surfaces, for the four magnetic cases, namely non-magnetic, ferromagnetic, and antiferromagnetic single and double layer structures. This is highly relevant for a mechanistic understanding of hydrogen embrittlement in austenitic steels, and we cover both, namely the earliest process, hydrogen adsorption, and the subsequent sub-surface diffusion. We focussed here mainly on the energetics of these processes with the overall aim to create minimum energy pathways through the iron lattice. It was found that the magnetic structure does not influence the lowest energy adsorption sites, but the surface plane does (4f for (100) and (110), and 3f for (111)), with adsorption energies of around 4 eV for almost all cases. Total energies barriers for the hydrogen to move into the sub-surface vary between 1.0 and 1.4 eV, and the bulk diffusion energies around 0.6 to 0.7 eV. These latter values agree very well with the recent experimental measurements of diffusion barriers in austenitic steels. While these energies are not too different for the various magnetic cases, the profile of the minimum energy pathways is noticeably different for all AFMD structures, and for all magnetic (110) surface cuts. Overall, we demonstrated the effects of surface planes and magnetic structure on the diffusion of hydrogen through austenitic Fe to atomistically model the early stages of hydrogen embrittlement. Structural components made from non-magnetic austenitic steels present a slightly higher total barrier to hydrogen adsorption and diffusion than all magnetic structures (with the only exception being the (100)AFM1 case which is 0.1 eV higher than the (100)NM case), and hence fabrication preferentially from non-magnetic γ-Fe would reduce the chances of hydrogen embrittlement. Equally, texturing steels such that grains which their (111) surface are exposed reduces the chances of hydrogen embrittlement as these present slightly higher total barriers than the respective (100) and (110) surfaces, with the only exception again being the (100)AFM1 case, which has a joint-2^nd^ highest total barrier of all 12 cases.

## Conflicts of interest

We declare no financial competing interests.

## Supplementary Material
